# Finger nodules with a papulovesicular hands and feet eruption: a complicated human Orf virus infection

**DOI:** 10.1186/s12879-024-08998-7

**Published:** 2024-01-17

**Authors:** Martina Salvi, Giorgio Tiecco, Luca Rossi, Marina Venturini, Simonetta Battocchio, Francesco Castelli, Eugenia Quiros-Roldan

**Affiliations:** 1 Department of Clinical and Experimental Sciences, Unit of Infectious and Tropical Diseases, University of Brescia-ASST Spedali Civili, Brescia, Italy; 2 Department of Clinical and Experimental Sciences, Section of Dermatology, University of Brescia, Brescia, 25123 Italy; 3 Department of Pathological Anatomy, ASST Spedali Civili, Brescia, Italy

**Keywords:** Orf, Erythema multiforme, Zoonosis, Viral infection, Nodule, Ecthyma contagiosum

## Abstract

**Background:**

Orf virus (ORFV) is the pathogen responsible for Orf, a zoonotic viral infection that can be spread to humans from sheep and goats. Here, we present a case of human Orf complicated by an immune-related reaction, to raise awareness of this under-recognized disease avoiding unnecessary investigations and overtreatment.

**Case report:**

A 51-year-old woman with no previous medical history presented with a one-week history of three asymptomatic swelling nodules with a grey necrotic center and red outer halo on her index finger. At physical examination there was also a pruritic papulovesicular eruption on her hands and feet. She reported a recent contact with a goat which had a similar nodular lesion in its mouth. A biopsy of the lesions was performed and a diagnosis of Orf complicated by widespread erythema multiforme was made based on the clinical and histopathological features. The lesions spontaneously resolved within the next 2 weeks.

**Conclusions:**

Orf is not very prevalent in our region, so we performed a biopsy of the lesion to guide us toward a diagnosis. However, we should remember that the diagnosis of ecthyma relies on clinical evaluation and epidemiological criteria.

## Background

Orf virus (ORFV) is the pathogen responsible for Orf, a zoonotic viral infection also known as *ecthyma contagiosum*. ORFV is an epitheliotropic DNA virus belonging to the *Parapoxvirus* genus that primarily infects animals, but transmission to humans can occur through direct or undirect contact with affected animals or contaminated raw meat [1]. At-risk populations mostly include people who work with sheep and goats [1]. The real prevalence of Orf lesions in both humans and livestock herds may be greatly underestimated, mainly because people living in rural settings often make a self-diagnosis and choose not to seek medical assistance [2].

Orf is still underreported and underrecognized as a result of the large differential diagnosis [3], the broad spectrum of possible immunological reactions, and the limited knowledge of several clinicians who are unfamiliar with this zoonotic viral disease [4]. A recently published systematic review suggested an increasing interest in these old and re-emerging diseases as most of the papers included were published after 2014 [5]; moreover, almost all the considered articles were published in dermatology journals, probably reflecting a lack of interest from other medical specialties, including infectious diseases [5].

Although Orf is considered an occupational disease, the infection may also occur in individuals with nonoccupational contact with sheep and goats [1], leading also to potential complications, including secondary bacterial infections, lymphadenopathy, lymphangitis, and secondary immunological manifestations such as erythema multiforme (EM), widespread papulovesicular eruption, Stevens–Johnson syndrome, or antibody-mediated hypersensitivity reactions such as blistering disorders [5]. To date, there is no efficient antiviral treatment to cure, or vaccine to prevent human Orf, which is usually a self-limiting disease. Several drugs have been reported in literature as an attempt to treat primary or secondary autoimmune lesions [5], such as imiquimod, cidofovir, interferon, acyclovir, and valacyclovir [5]. However, no antibiotics or steroids are generally required in these auto-resolving manifestations.

Here, we present a case report of an ORFV infection complicated by an immune-related reaction to raise awareness of this under-recognized disease.

## Case presentation

**Fig. 1:**
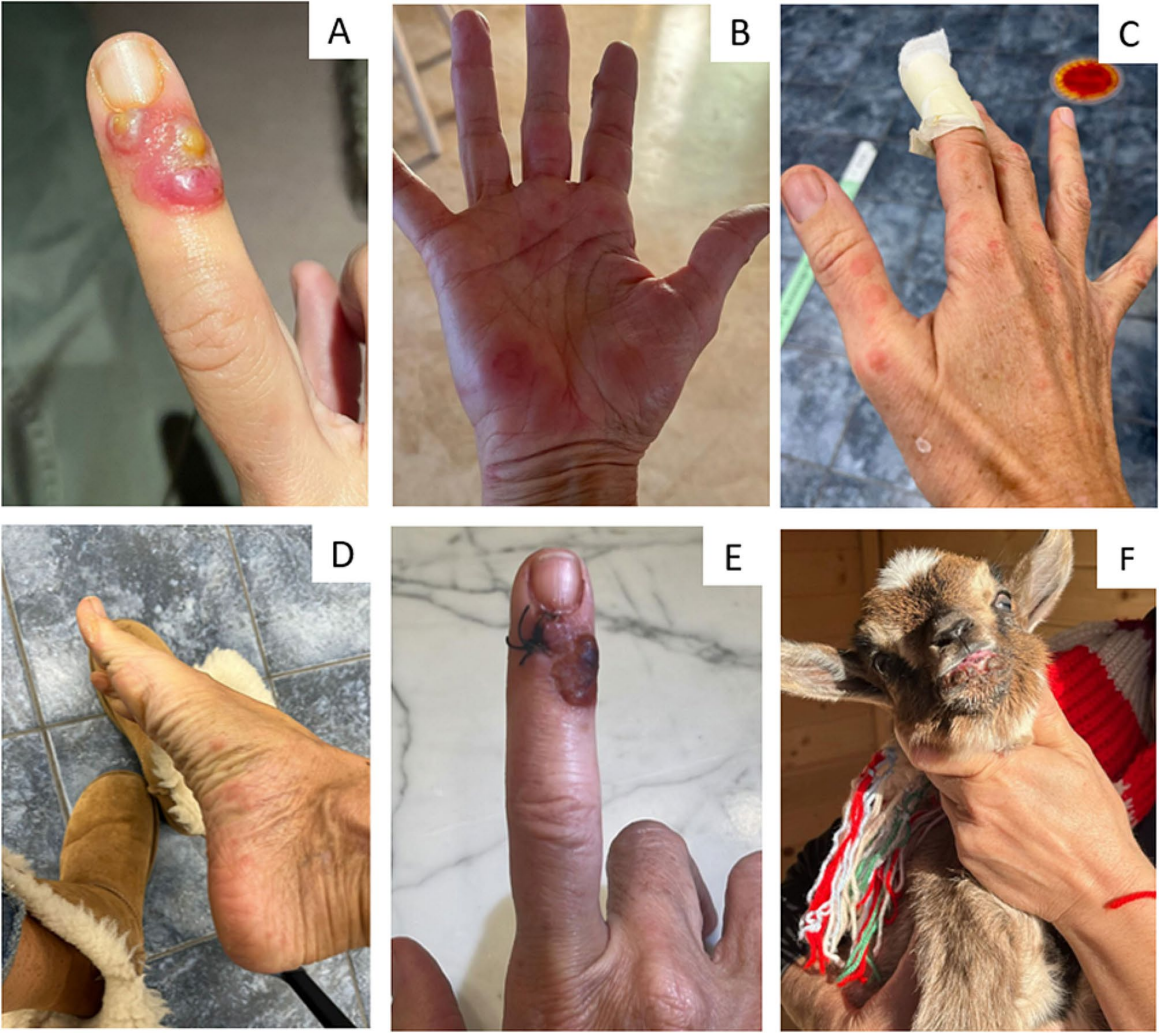
*Ecthyma contagiosum* and an autoimmune complication: asymptomatic swelling nodules with a necrotic center and a red outer halo (**A**), pruritic papulovesicular eruption on hands and feet (**B**-**C**-**D**), primary lesion biopsy (**E**) mucosal nodule in the goat touched by the patient(**F**)

**Fig. 2:**
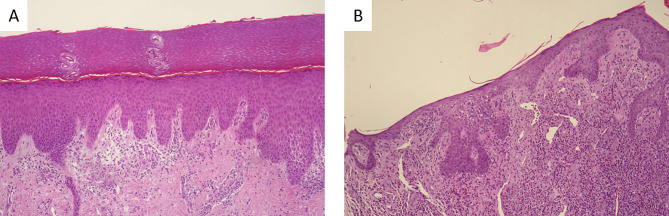
Histopathology: vesicular dermatitis with eosinophils (**A**), urticarial dermatitis (**B**)

In December 2022, a 51-year-old woman came to our attention in a Northern Italy Clinic for a one-week history of three unpainful lesions on her right index finger (Fig. 1A). At physical examination, the lesion appeared as three swelling nodules with a grey necrotic center and red outer halo (Fig. 1A). Moreover, a pruritic papulovesicular eruption was present on her hands and feet, which was the real reason for medical consulting (Fig. 1B and C-1D). At the beginning, the rash appeared like round erythematous papules and then evolved into target lesions with a central area and dark red inflammatory zone surrounded by a pale ring of edema (Fig. 1C and D). The woman reported that the rash appeared a few days after the initial lesions on the finger. No relevant conditions were present in the patient’s past medical history. Particularly, she did not take any drugs chronically. Blood tests revealed inflammation markers within normal ranges, as shown in Table 1, with a white cell count of 3.07 g/L (reference range 4–10 g/L), and C-reactive protein < 0.05 mg/L (reference range < 5 mg/L). The Quantiferon TB-gold test was performed, and it turned out negative; only a slight modification of liver function was visible. A biopsy was performed on one finger nodule (Fig. 1E) and on a papulovesicular lesion (Fig. 1D). Microscopic examination of the primary lesion showed intraepidermal vesiculation with irregular hyperplasia and a mixed inflammatory cell infiltrate (Fig. 2A). The papulovesicular eruption was histologically compatible with an urticarious reaction (Fig. 2B). As a matter of fact, when asked, the woman reported sporadic contact with a goat that had a similar erythematous nodule in her mouth during a holiday in Sicily (Italy), touching the animal’s mouth with bare hands while feeding it (Fig. 1F). Goat was diagnosed with ORFV infection, but the patient was not warned about the possible hypersensitive reaction during the next days. No treatment was needed for the recovery of the lesions, which spontaneously resolved within the next 6 weeks. Based on these findings, ORFV infection with an autoimmune widespread erythema multiforme was diagnosed.

**Table 1:** Report of admission lab tests. WBC: white blood cells; RBC: red blood cells; HGB: haemoglobin; HCT: haematocrit; MCV: mean corpuscular volume; MCHC: mean corpuscular haemoglobin concentration; RDW: red cell distribution width; PLT: platelets; MPV: mean platelet volume; ESR: erythrocyte sedimentation rate; GFR: glomerular filtration rate; AST: aspartate aminotransferase; ALT: alanine transaminase; GGT: gamma-glutamyl transferase; CRP: C-reactive protein

**WBC**	
Neutrophils	3.07 × 10^9/L (57.9%)
Lymphocytes	1.19 × 10^9/L (22.6%)
Monocytes	0.53 × 10^9/L (10%)
Eosinophils	0.47 × 10^9/L (8.9%)
Basophils	0.03 × 10^9/L (0.6%)
**RBC**	4.29 × 10^12/L
**HGB**	136 g/L
**HCT**	41.8%
**MCV**	97.4 fL
**MCHC**	32 pg
**RDW**	325 g/L
**PLT**	208 × 10^9/L
**MPV**	11.4 fL
**ESR**	5 mm/h
**Creatinine**	0.77 mg/dL
**GFR**	< 90 mL/min/1.73m2
**AST**	61 U/L
**ALT**	70 U/L
**GGT**	14 U/L
**CRP**	< 0.05 mg/L
**Quantiferon TB-gold**	Negative

## Discussion and conclusions

Orf is endemic in sheep and goats worldwide. Human infections typically occur in case of occupational risks, primarily affecting shepherds, veterinarians, and people who work in abattoirs [6]. Clinical symptoms in animals begin with the appearance of erythematous papules (3–5 mm in diameter) around the lips, nares, and buccal cavity. Similar lesions might be also detected in more severe cases on the epidermis, breast, eyes, foot, or vulva. After a few days, these lesions grow up taking on a verrucose appearance on the tongue and tooth pad before becoming ulcerated and producing an exudate that causes extremely contagious pustules and scabs [7]. Different studies pointed out that ORFV survives on animal wool for around a month after the lesions are solved.

The virus is characterized by a particular resistance to environmental inactivation and it has been found on dried crusts even after several months to years [2]. Our patient denied any occupational exposure to animals, however a sporadic contact with an infected goat occurred during her holidays. Only a few cases of human-to-human transmission have been reported [8]. Human infection typically manifests as a single papule or nodule 3 to 7 days after inoculation. It progresses through 6 stages before spontaneous remission occurs 6 to 8 weeks later. Usually on a finger or hand, the initial lesion is a tiny, hard, reddish-blue unpainful papule at the site of ORFV entrance. The papule transforms into a bulla or pustule that bleeds and quickly forms a central crust. Frequently the lesion undergoes bacterial infection [2]. In our case report, as in the case reported by Yanfei Li [9], the patient manifested a single unpainful nodule on her finger that evolved in crust without superposition of bacterial infection. No fever, malaise, or lymphadenopathy were assessed.

Current studies suggest that ORFV can cause a variety of hypersensitivity reactions being erythema multiforme (EM) the most common immunological consequence recorded [10], which typically appears before the primary lesions have completely healed and generally 3 weeks following the onset of Orf [1]. EM has been related to Orf in 7–18% of patients [1], but mechanisms of Orf-induced autoimmune reactions are still unclear and systemic dissemination of the virus has not yet been proven [11]. It has been established that ORFV infects keratinocytes and dermal fibroblasts, inducing apoptosis [12]. According to recent theories, immune-mediated problems can result from molecular mimicry of host proteins and from alterations of proteins that form the basement membrane, which increases immunogenicity [11, 12]. According to a recently published systematic review including all published articles regarding Orf infections complicated by secondary immune-mediated reactions in humans, EM was the most frequently reported immunological complication, always appearing before the complete resolution of primary lesions, as happened in our case report [5]. However, this hypersensitivity reaction is not Orf-exclusive, as it has been associated with other infectious diseases being herpes simplex virus (HSV) the leading cause [13]. Whereas the mechanism of HSV-EM has been extensively studied and well established, the Orf-induced autoimmune diseases are still unknown [5].

As regards Orf diagnosis in humans, the distinctive lesion and the history of exposure are typically used to make a clinical diagnosis when laboratory tests are not available. According to the literature, only a limited number of patients undergo a biopsy [14]. For ORFV detection, several molecular-based diagnostic methods that target specific ORFV DNA fragments are extensively employed in veterinary medicine [15]. Moreover, although the diagnostic methods are available, the diagnosis of Orf is primarily clinical and epidemiological and does not require further analysis. For this reason, in the present clinical case, molecular diagnostic methods were not performed.

As differential diagnosis, several clinical conditions should be considered as pyoderma, herpetic whitlow, cowpox, monkeypox, pseudocowpox (milker’s nodule), cat-scratch disease, anthrax, tularaemia, primary inoculation TB, atypical mycobacteriosis, syphilitic chancre, sporotrichosis and keratoacanthoma [16]. Because of the global spread of the monkeypox virus and its clinical similarity with Orf, an accurate anamnesis is essential, especially when laboratory tests are not available and must include a collection of exposures to possible zoonoses and other risk factors (such as sexual contact) [17, 18]. Monkeypox and Orf lesions can be similar, but ORFV infection can be suspected because of contact with infected animals, localized lesions in the point of contact (usually hands) and the absence of systemic symptoms like fever, which is common in Monkeypox. Systemic symptoms like lymphadenitis fever, headaches, and malaise are often associated with pathologies considered in the differential diagnosis such as Monkeypox, cat-scratch disease, tularaemia, anthrax, and cowpox virus [18–22].

In immunocompetent individuals, ORF lesions often spontaneously resolve within a maximum of 8 weeks, as reported in our case study. However, several pharmacological strategies, including imiquimod, cidofovir, interferon, acyclovir, and valacyclovir, as well as surgical excisions are described in the literature for ORF treatment despite its self-limiting nature [2]. Immunological reactions are treated in a greater percentage of cases, suggesting that these systemic manifestations cause a more significant clinical impact than the localized initial lesions. In fact, most patients usually consult a doctor only when complications occur, as in the case of the patient who came to our attention. Topical or systemic corticosteroids are typically used, based on the suspect immune-mediated nature of the symptoms [23]. More studies are required to examine the potential efficacy of imiquimod, as some reports described a quick resolution of both Orf and immune-mediated reactions after applying it exclusively to initial lesions [24].

Here we describe a case of ORFV infection complicated by an immune-mediated reaction in a region where it is very uncommon to diagnose this zoonotic disease. Since the condition is not very prevalent in our region, we chose to perform a biopsy of the lesion to guide us toward a diagnosis together with clinical evaluation and epidemiological criteria. The final aim of this case report is to raise concern in regards to the primary presentation and its potential complications avoiding unnecessary diagnostic tests and overtreatment.

## Data Availability

Biological and microbiological data have been made available to the editors. For further information please contact the corresponding author.

## Abbreviations

ORFV: Orf virus
EM: Erythema multiforme
WBC: White blood cells
RBC: Red blood cells
HGB: Haemoglobin
HCT: Haematocrit
MCV: Mean corpuscular volume
MCHC: Mean corpuscular haemoglobin concentration
RDW: Red cell distribution width
PLT: Platelets
MPV: Mean platelet volume
ESR: Erythrocyte sedimentation rate
GFR: Glomerular filtration rate
AST: Aspartate aminotransferase
ALT: Alanine transaminase
GGT: Gamma-glutamyl transferase
CRP: C-reactive protein
HSV: Herpes simplex virus
PCR: Polymerase chain reaction
RPA: Recombinase polymerase amplification
RAA: Recombinase aided amplification

## References

[1] Bergqvist C, Kurban M, Abbas O. Orf virus infection. Rev Med Virol. 2017;27(4). doi: 10.1002/rmv.1932. Epub 2017 May 8. PMID: 28480985. 10.1002/rmv.1932 28480985

[2] Kassa T (2021). A review on human orf: a neglected viral zoonosis. Res Rep Trop Med.

[3] Hosamani M, Scagliarini A, Bhanuprakash V, McInnes CJ, Singh RK. Orf: an update on current research and future perspectives. Expert Rev Anti Infect Ther. 2009;7(7):879– 93. 10.1586/eri.09.64. PMID: 19735227. 10.1586/eri.09.64 19735227

[4] Georgiades G, Katsarou A, Dimitroglou K. Human ORF (ecthyma contagiosum). J Hand Surg Br. 2005;30(4):409– 11. 10.1016/j.jhsb.2005.03.010. PMID: 15936127. 10.1016/j.jhsb.2005.03.010 15936127

[5] Rossi L, Tiecco G, Venturini M, Castelli F, Quiros-Roldan E (2023). Human Orf with Immune-mediated reactions: a systematic review. Microorganisms.

[6] Alinejad F, Momeni M, Keyvani H, Faramarzi S, Mahboubi O, Rahbar H (2018). Introduction to a case of orf disease in a burn wound at Motahari Hospital. Ann Burns Fire Disasters.

[7] Onyango J, Mata F, McCormick W, Chapman S. Prevalence, risk factors and vaccination efficacy of contagious ovine ecthyma (orf) in England. Vet Rec. 2014;175(13):326. 10.1136/vr.102353. Epub 2014 Jul 4. PMID: 24996900. 10.1136/vr.102353 24996900

[8] Wang R, Wang Y, Liu F, Luo S. Orf virus: A promising new therapeutic agent. Rev Med Virol. 2019;29(1):e2013. doi: 10.1002/rmv.2013. Epub 2018 Oct 28. PMID: 30370570. 10.1002/rmv.2013 30370570

[9] Li Y, Li X, Wu S, Mu Q, Ma L. Rapid evolving cutaneous lesions on the hand: a diagnostic challenge. Travel Med Infect Dis 2023 May-Jun;53:102572. 10.1016/j.tmaid.2023.102572. Epub 2023 Apr 7. PMID: 37030520. 10.1016/j.tmaid.2023.102572 37030520

[10] Johannessen JV, Krogh HK, Solberg I, Dalen A, van Wijngaarden H, Johansen B. Human orf. J Cutan Pathol. 1975;2(6):265– 83. 10.1111/j.1600-0560.1975.tb00179.x. PMID: 1219046. 10.1111/j.1600-0560.1975.tb00179.x 1219046

[11] Zuelgaray E, Salle de Chou C, Gottlieb J, Battistella M, Vignon-Pennamen MD, Bagot M, Guibal F, Bouaziz JD (2018). Human orf complicated by epidermolysis bullosa acquisita. Br J Dermatol.

[12] Schneider LE, Protschka M, Müller U, Muhsen M, Magin TM, Anderegg U, Saalbach A, Büttner M, Alber G, Siegemund S. Orf virus infection of human keratinocytes and dermal fibroblasts: Limited virus detection and interference with intercellular adhesion molecule-1 up-regulation. Exp Dermatol. 2019;28(2):142–151. 10.1111/exd.13861. PMID: 30554456. 10.1111/exd.13861 30554456

[13] Hafsi W, Badri T, Erythema M. 2023, *StatPearls [Internet]. Treasure Island (FL)*.

[14] Pang F, Long Q (2023). Recent advances in diagnostic approaches for Orf virus. Appl Microbiol Biotechnol.

[15] Wang Y, Yang K, Bai C, Yin D, Li G, Qi K, Wang G, Li Y. Development of a SYBR Green I real-time PCR for the detection of the Orf virus. AMB Express. 2017;7(1):21. 10.1186/s13568-016-0322-9. Epub 2017 Jan 7. PMID: 28063148; PMCID: PMC5218949. 10.1186/s13568-016-0322-9 PMC5218949 28063148

[16] Tiecco G, Degli Antoni M, Storti S, Marchese V, Focà E, Torti C, Castelli F, Quiros-Roldan E. A 2021 Update on Syphilis: Taking Stock from Pathogenesis to Vaccines. Pathogens. 2021;10(11):1364. 10.3390/pathogens10111364. PMID: 34832520. 10.3390/pathogens10111364 PMC8620723 34832520

[17] Caldas JP, Valdoleiros SR, Rebelo S, Tavares M (2022). Monkeypox after Occupational Needlestick Injury from Pustule. Emerg Infect Dis.

[18] Cavalieri C, Dupond AS, Ferrier-Rembert A, Ferraris O, Klopfenstein T, Zayet S (2023). Orf Nodule with Erythema Multiforme during a Monkeypox Outbreak, France, 2022. Emerg Infect Dis.

[19] Kamstra JI, van der Meij EH, de Visscher JGAM. Kattenkrabziekte [Cat scratch disease]. Ned Tijdschr Tandheelkd. 2021;128(1):21–27. Dutch. 10.5177/ntvt.2021.01.20049. PMID: 33449053. 10.5177/ntvt.2021.01.20049 33449053

[20] Morand A, Delaigue S, Morand JJ. Review of poxvirus: emergence of monkeypox. Med Sante Trop. 2017;27(1):29–39. English. 10.1684/mst.2017.0653. PMID: 28406414. 10.1684/mst.2017.0653 28406414

[21] Kamal SM, Rashid AK, Bakar MA, Ahad MA (2011). Anthrax: an update. Asian Pac J Trop Biomed.

[22] Marckmann D, Frasnelli A (2020). Milker’s nodule (pseudocowpox) in a female patient following a calf bite. Dtsch Arztebl Int.

[23] Erbağci Z, Erbağci I, Almila Tuncel A. Rapid improvement of human orf (ecthyma contagiosum) with topical imiquimod cream: report of four complicated cases. J Dermatolog Treat. 2005;16(5–6):353-6. 10.1080/09546630500375734. PMID: 16428161. 10.1080/09546630500375734 16428161

[24] Soares A, Sokumbi O (2021). Recent updates in the Treatment of Erythema Multiforme. Med (Kaunas).

